# Clarifying the Mechanisms of Antidepressants

**DOI:** 10.1093/ijnp/pyv104

**Published:** 2015-09-12

**Authors:** Don M. Tucker

**Affiliations:** University of Oregon and Electrical Geodesics, Inc.

Commentary on Domschke, et al., Magnetoencephalographic correlates of emotionalprocessing in major depression before and after pharmacological treatment.

Understanding the therapeutic effects of psychotropic medication has typically been approached, reasonably enough, with an analysis of the effects on brain chemistry. For example, the effective antidepressant mirtazapine is thought to affect both norepinephrine and serotonin neuromodulators, both of which are important to mood disorders such as major depression.

However, understanding the full neuropsychopharmacology of a therapeutic agent requires an analysis of the multiple levels of neural self-regulation that are involved in the disorder and that are affected by the drug. It has long been apparent that if the mechanism of drug action were simply a correction of a biochemical imbalance, then the therapeutic effect would be rapid, limited only by the pharmacokinetics (Cooper et al., 2002). Instead, antidepressants typically require weeks of continued administration before they achieve a therapeutic effect.

Although the complexity of drug action is familiar to most psychopathology researchers, clinicians are typically provided with only a superﬁcial training on the neurophysiological and neuropsychological mechanisms for both psychopathology itself and the therapeutic effects of drugs. The result of this limited training is that a patient with depression, for example, is often told by the physician they have a “biochemical imbalance” that will be corrected by drug therapy.

Not only is this explanation scientiﬁcally incorrect, it fails to recognize that the chronic changes in clinical depression involve a predictable set of neural mechanisms, including not only impairment of hedonic arousal and reward sensitivity that follow from brainstem neuromodulator changes (Depue and Morrone-Strupinsky, 2005), but blunted right hemisphere processing of affective material and changes in fronto limbic mechanisms of self-regulation (Mayberg, 2007). The chronic alterations of fronto limbic activity are particularly important, because they appear to reﬂect a disordered pattern of affective self-regulation that may be responsible for both the anhedonia and neuromodulator changes (Tucker and Luu, 2007). The fact that improved psychological self-regulation, such as with cognitive therapy, can be as effective as drug therapy (Curry et al., 2006) provides important evidence that depression must be understood to involve multiple levels of neuropsychological function. In a similar way, the therapeutic neuropsychopharmacological effects should be understood in relation to the full set of interrelated self-regulatory mechanisms—psychological, fronto limbic, and neuromodulator control—that will determine improved clinical outcome.

The report in this issue by Domschke et al. presents an important demonstration that neurophysiological measures during emotional processing can help clarify not only the self-regulatory abnormality in clinical depression, but also the changes in neural and psychological self-regulation that occur with successful drug therapy.

The magnetoencephalographic (MEG) measures were obtained from the whole head, allowing examination of multiple cerebral regions and networks simultaneously. Before treatment, depressed persons showed a decreased response to emotional pictures in the right temporoparietal area compared with matched healthy controls. The greater the severity of depression, the more the response to pictures was suppressed in this right posterior region. Importantly, this effect was apparent within 150 milliseconds of the presentation of the picture, consistent with the previous work from the Junghofer laboratory showing that emotional responses may manifest very early in the perceptual process.

The MEG methodology of this study is particularly relevant for characterizing the time course of the neural response. Whereas PET and fMRI ﬁndings have been important in showing regional disorders in depression, only EEG and MEG measures allow this close inspection of the time course of the abnormal brain activity. Whatever the neural mechanism that causes the depressed person to be less responsive to the emotional material, it appears to operate early, as a fundamental aspect of perception, particularly in the right cerebral hemisphere.

The nature of this mechanism was further clariﬁed by the simultaneous measures of frontal lobe activity. The greater the person’s depression, the greater was the MEG source activity in the right dorsolateral frontal lobe. Given ﬁndings from fMRI studies suggesting the right frontal lobe is involved in regulating positive affect and that an exaggeration of this regulation is seen in depression (Light, Heller et al. 2011), the right frontal lobe activity in the present study may suggest an exaggerated pattern of fronto limbic self-regulation. Importantly, the increased right frontal activity was speciﬁc to the emotionally positive pictures, and this effect was greater at higher Hamilton depression scores, suggesting it was associated with greater symptom severity.

Following 4 weeks of mirtazapine treatment, the patients showed an apparent normalization of the right temporoparietal response, with the response of patients no longer different from controls. Interestingly, the dorsolateral frontal measures showed both groups had stronger engagement on the left hemisphere, but the patients increased their response in the right dorsolateral frontal region, whereas controls decreased in this area.

How do we interpret these multiple cerebral and cognitive effects in relation to the proximal mode of action of mirtazapine, apparently affecting noradrenergic and serotonergic modulation? An important clue may be the hemispheric asymmetry of these neuromodulators, particularly in humans. The major (dorsal) noradrenaline (norepinephrine) projection system, for example, appears to be right-lateralized, extending to the frontal pole before projecting caudally to the entire hemisphere. An important hypothesis may be that the pathological self-regulation in chronic depression involves a normal (right-lateralized) fronto limbic inhibition over positive affect and hedonic responses that becomes chronically ﬁxed. The fronto cortical regulation must affect the limbic network but also the brainstem neuromodulators that regulate the mood of positive affect.

A neuropsychopharmacological therapy such as mirtazapine may serve to reverse the neuromodulator state and thereby restore a certain balance to the multiple midbrain, limbic, and neocortical networks, particularly in the emotionally signiﬁcant right hemisphere. By characterizing the relevant functional brain activity in studies such as the Domschke et al. work, it may be possible not only to understand the neural complexity of human affective self-regulation but also to design clinical interventions that optimize pharmacological with psychological therapy. Cognitive therapy, for example, may have longer lasting effects than drug therapy for depression (Rohde, Silva et al., 2008). Yet severely depressed patients may not have sufﬁcient initiative to engage in cognitive therapy effectively, unless they receive a sufﬁcient pharmacotherapy ﬁrst. If the right hemisphere’s processing of social and affective information is blunted in the depressed state, the person may be incapable of applying the disciplines of cognitive therapy to achieve psychological improvement. By understanding the speciﬁc neural mechanisms of depression and how they change with neuropsychopharmacological treatment, it may be possible to design coordinated interventions for the multiple levels of neuropsychological self-regulation that are required for lasting improvement in life skills.
