# Erratum

**DOI:** 10.1093/ijnp/pyw010

**Published:** 2016-02-23

Tucker DM. Clarifying the Mechanisms of Antidepressants. Int J Neuropsychopharmacol 19(1). doi: 10.1093/ijnp/pyv104.

In Volume 19, Issue 1 of *The International Journal of Neurop sychopharmacology*, the Focus Paper DOI: 10.1093/ijnp/pyv104 was published without its related research article. The research article is:

Domschke K, Zwanzger P, Rehbein MA, Steinberg C, Knoke K, Dobel C, Klinkenberg I, Kugel H, Kersting A, Arolt V, Pantev C, Junghofer M. Magnetoencephalographic Correlates of Emotional Processing in Major Depression Before and After Pharmacological Treatment. Int J Neuropsychopharmacol. doi: 10.1093/ijnp/pyv093.

It will publish in Volume 19, issue 2.

